# Paliperidone palmitate 6-month formulation for the treatment of schizophrenia: a 14-month follow-up study

**DOI:** 10.1192/j.eurpsy.2024.1565

**Published:** 2024-08-27

**Authors:** S. L. Romero Guillena, H. Marín Mateos, G. Rodriguez Menendez

**Affiliations:** Psychiatry, U.S.M.C Carmona. UGC Mental Health Virgen Macarena, Seville, Spain

## Abstract

**Introduction:**

Relapse prevention is critical because psychopathology and functionality can worsen in patients with schizophrenia because the repeated episodes and we have strong evidence of antipsychotics efficacy for relapse prevention, but nonadherence rates in patients with schizophrenia are very high, even in comparison with other illness.

There is extensive clinical trial evidence for the use of paliperidone palmitate 1-month (PP1M) and paliperidone palmitate 3-month (PP3M) formulations for maintaining treatment continuity and preventing relapses and risk of hospitalizations in patients with schizophrenia. (Najarian et al. Int J Neuropsychopharmacol 2022; 25(3) 238-251). Paliperidone palmitate 6-month (PP6M) formulation is a presentation that provides a dosing interval of once every six months.

**Objectives:**

The principal aim of this study was to evaluate the effectiveness, safety, and tolerability of the PP6M in patients with non-acute schizophrenia on an outpatient basis

**Methods:**

Methods: Sample: 22 patients diagnosed with schizophrenia (DSM 5 criteria) that started treatment with PP6M after being stabilized with PP1M (N:10) or PP3M (N:12) (the treatment dose was not changed in the four months before study inclusion)

Bimonthly, the following evaluations were performed during a follow-up period of 14 months:

The Clinical Global Impression-Schizophrenia scale (CGI-SCH)

Treatment adherence, concomitant medication, adverse events and the number of hospitalizations and emergency visits

Efficacy values: Percentage of patients who remained free of admissions at the end of 14months of follow-up.

Other evaluation criteria: Percentage of patients who never visited the emergency department at the end of 14 months of follow-up, average change from baseline visit to the final evaluation as assessed by score obtained on the following scale: GSI-SCH, treatment adherence rate and tolerability.

**Results:**

The percentage of patients who remained free of admission at the end of the 14 months follow-up was 90% in the total sample, 83% in the PP3M pre-treatment group and 100% in the PP1M pre-treatment group.

The percentage of patients who never visited the emergency department at the end of 14 months follow-up was: 81% in the total sample, 75% in the PP3M pre-treatment group and 90% in the PP1M pre-treatment group.

At the end of the study, a mean change of +0.12 (±0.11) on the ICG-SCH-SI scale in the total sample, +0.25 (±0.21) in the PP3M pre-treatment group and 0 in the PP1M pre-treatment group.

The treatment persistence rate at the 14 month of follow-up was 100% in the total sample.

Treatment was well tolerated, and no safety-related adverse events were collected. There were no tolerability-related withdrawals from treatment.

**Conclusions:**

In our study, we found that long-term treatment with paliperidone palmitate 6-month formulation is effective and well tolerated in clinical practice conditions.

**Disclosure of Interest:**

None Declared

